# Analysis of Asymmetry of the Forces Applied on the Lower Limb in Subjects with Nonspecific Chronic Low Back Pain

**DOI:** 10.1155/2014/289491

**Published:** 2014-07-01

**Authors:** Maryam Hassan Zahraee, Mohammad Taghi Karimi, Javid Mostamand, Francis Fatoye

**Affiliations:** ^1^Musculoskeletal Research Center, Isfahan University of Medical Sciences, P.O. Box 81745-164, Isfahan, Iran; ^2^Department of Health Professions, Manchester Metropolitan University, UK

## Abstract

*Objective*. Several studies have investigated asymmetry and loading patterns in different spine pathologies, motor disorders, and other conditions; there is a lack of knowledge on these aspects in chronic low back pain (CLBP). The aim of this study was to analyse asymmetry and loading patterns in patients with nonspecific chronic low back pain (NCLBP) compared to normal individuals, during walking. *Method*. Forty participants (20 healthy subjects and 20 patients with NCLBP) participated in the study. Asymmetry of the force was measured based on the Asymmetry Index (ASI). The difference in the mean values of all data between the two groups was examined using the independent *t*-test. *Results*. The mean value of the first peak of ground reaction force of normal subjects was 1.02 ± 0.0354 N/BW compared to 1.038 ± 0.099 N/BW in NCLBP patients (*P* = 0.25) and 0.1004 ± 0.036 N/BW mediolateral force applied on the leg in normal subjects compared to 0.089 ± 0.022 N/BW in NCLBP patients (*P* = 0.214). The Asymmetry Index (ASI) of the first peak of vertical force was 2.59% ± 1.89% and 3.88% ± 2.94% for NCLBP and normal subjects, respectively, *P* = 0.2. *Conclusion*. Therefore, it can be concluded that NCLBP subjects follow avoidance-endurance model without any limitation during walking.

## 1. Introduction

Low back pain (LBP) remains a prevalent and persistent problem that is associated with functional limitations and participation restrictions [1–3]. Evidence suggests that increased activity level such as walking is an effective way of managing patients with LBP; hence, therapeutic exercise programmes have been recommended for individuals with this condition [4–8]. While some people with LBP remain active, others have difficulty doing so due to physical, psychological, and social reasons, and this can contribute to distress and disability, increasing the economic cost of chronic LBP.

Walking is a complex task that involves the coordination of the muscular system to simultaneously produce and sustain a variety of multidirectional forces around each joint and with the ground, that is, ground reaction forces (GRFs) and maintaining balance in an upright posture [9]. It is evident that LBP has a profound impact on gait [10, 11]. Clinicians commonly recommend gait reeducation programme for people with LBP as part of their rehabilitation. Therefore, it is important to have a good understanding of how LBP affects walking so as to develop more appropriate walking programs for this population. However, individuals with LBP generally have walking problems, which, in turn, may reduce their willingness to participate in exercise programs [12].

As a valuable tool for the understanding of motion disorders and treatment outcomes, clinical gait analysis is based on the quantification and evaluation of deviations from normal values [12–14]. Kinetic, as a gait parameter, reflects the cause of movement, and therefore the forces, power, and energy that affect the manner in which an individual moves. Also, GRFs measured with force plates imbedded in the ground or treadmill refer to the forces that act on the body throughout the stance phase. The symmetry of motions and also the forces applied on the legs have being used to determine the severity of disease and also the effectiveness of treatment interventions [15–18]. In pathological gait a noticeable asymmetry has been recorded between the affected and unaffected lower limbs [18, 19]. Hence, asymmetry may be a significant factor of pathology. Therefore, it is important that this parameter should also be evaluated as part of a gait analysis. Although there are several studies on asymmetry and loading patterns in different spine pathologies such as scoliosis [19], motor disorders, cerebral palsy [20, 21], and other conditions such as lower limb amputations [17], there is a lack of knowledge on the asymmetry of gait parameters in patients with CLBP.

These findings may help to provide a good understanding of the possible effects of CLBP on gait parameters. Ultimately, they can be used for identifying any abnormality and to inform appropriate treatment plan for patients with this condition. The present study, therefore, aimed at analyzing asymmetry and loading patterns of legs in patients with CLBP compared with their healthy counterparts. The main hypothesis associated with this study was that there was no asymmetry of applied forces in subjects with CLBP.

## 2. Materials

Forty participants (20 healthy subjects and 20 patients with NCLBP) participated in the study. Healthy subjects were selected from the staff members of Rehabilitation Faculty of Isfahan University of Medical Sciences and were matched with patients based on age and height.  Table 1 shows the characteristics of both groups.

Patients were recruited from the subjects referred to Physical Therapy clinic of the faculty, based on inclusion/exclusion criteria shown in Table 2. Ethical approval was obtained from the Ethics Committee of Isfahan University of Medical Sciences. Informed written consent was obtained from all the participants. The participants were asked to walk along the gait lab path with a comfortable speed, from which 5 successful trials were selected. A Kistler force plate was used to record the forces applied on leg during walking. The data of the force plate was collected with a frequency of 120 Hz and was filtered with a Butterworth low pass filter with a cut-off frequency of 10 Hz. The peaks of the vertical force, anteroposterior force, and mediolateral force applied on the right and left legs were used for final analysis. Moreover, the symmetry of the force applied on legs in normal and NCLBP patients was determined, according to Asymmetry Index measure. The asymmetry was measured based on the Asymmetry Index (ASI) described by Herzog et al. [22], based on the following equation:

(1) $$\begin{array}{c} \text{ASI}=\;\frac{X\;\text{right}-X\;\text{left}}{\left(X\;\text{right}+X\;\text{left}\right)/2}, \end{array}$$

in which *X* is the value of the parameter. An ASI value of zero indicates that there is perfect symmetry for the particular gait variable. Shapiro-Wilk test revealed that the data was normally distributed; hence, the independent* t*-test was used to compare the two groups.

## 3. Results

Table 3 shows the mean values of spatiotemporal gait parameters of normal subjects and those with NCLBP. The mean values of walking speed in healthy participants and patients with NCLBP were 9.53 ± 0.99 and 9.2 ± 1.3 cm/s, respectively, *P* = 0.245. There was no significant difference in the mean values of spatiotemporal gait parameters between health participants and patients with NCLBP (*P* > 0.05).

Table 4 summarizes the mean values of forces applied on the leg in three directions. The mean value of the first peak of ground reaction force of normal subjects was 1.02 ± 0.0354 N/BW compared to 1.038 ± 0.099 N/BW in patients with NCLBP (*P* = 0.25). There was a significant difference in the vertical force applied on the leg during push-off phase (third peak) between healthy subjects and patients with NCLBP groups (*P* = 0.038). The mediolateral force applied on the leg in healthy subjects was 0.1004 ± 0.036 N/BW compared to 0.089 ± 0.022 N/BW in patients with NCLBP (*P* = 0.214).

Table 5 shows the ASI in healthy subject and patients with NCLBP groups during walking on a level surface. The ASI of walking speed in healthy subjects was 5.03 ± 4.6% compared with 3.8 ± 3.4% in patients with NCLBP (*P* = 0.3). The ASI of the first peak of vertical force was 2.59 ± 1.89% and 3.88 ± 2.94% in patients with NCLBP and healthy participants, respectively, *P* = 0.2. The ASI of the second and third peaks of vertical ground reaction force varied between 2.4 to 2.48 in healthy subjects compared to 2.4 to 17.12 in patients with NCLBP.

## 4. Discussion 

**Figure 1:**
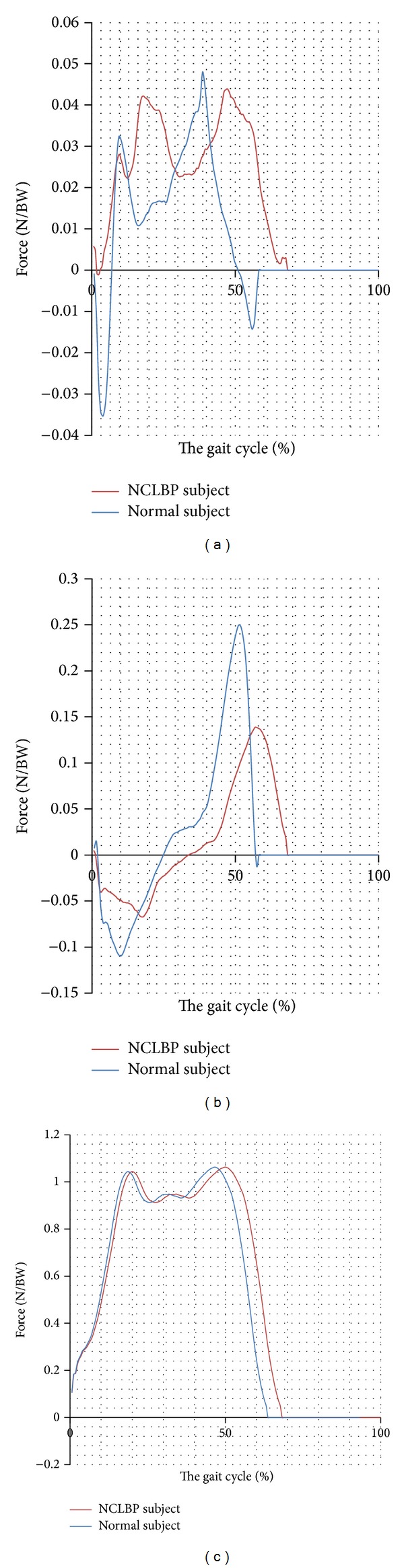
The mediolateral (a), anteroposterior (b), and vertical forces (c) applied on the leg of a normal and a NCLBP subject.

Approximately 80% of adult population experience LBP at any time during their life [3]. It has been shown that nearly 90% of all patients with LBP suffer from a pain with an unknown origin, which is defined as nonspecific low back pain (NCLBP) [23, 24]. To date, there is no information regarding the loads applied on the leg in patients with NCLBP. Moreover, the symmetry of loads applied on limb in patients with NCLBP is currently unknown. Therefore, the aim of this research was to evaluate the loads and symmetry of loading applied on the leg in patients with NCLBP., Figure 1 show The mediolateral, anteroposterior, and vertical forces applied on the leg of a normal and a NCLBP subject.

The mean values of the loads applied on the dominant side of normal subjects and those with NCLBP are shown in Table 4. As it can be seen, there was no difference between the peaks of most of the forces applied on the leg. The only difference was related to the vertical force of push-off phase. This suggests that NCLBP patients walked nearly the same as normal subjects, as there was no difference between the spatiotemporal gait parameters of both groups. This finding contradicts that reported by Khodadadeh et al., 1998, and Keefer and Hill, 1985, who observed that patients with LBP tend to walk slower than normal subjects as observed in previous studies. The reason for our findings is not obvious, as clinical gait assessments such as functional gait assessment or Tenetti were not carried out to verify if gait was actually affected by back pain or not in these patients.

However, the main reason for decrease in vertical force applied on the leg during push-off phase may be related to the angle of the joints in this stage of gait cycle. If the flexion angle of knee or hip joint decreases, then the loads applied on the leg will decrease as well (due to decrease in displacement of center of gravity in downward direction) [9, 25]. Therefore, it can be concluded that the decrease in loads of leg in this stage may be related to the kinematic of the joints. However, it should be emphasized that there was no asymmetry in the peaks of the vertical force applied on the leg during push-off between normal subjects and those with NCLBP. It means that the reduction of this force occurred in both legs simultaneously. In addition, the back muscles are usually active during the stance phase to counteract forward momentum produced by the lower limb (Nelson et al., 1995). The higher the push-off force is, the more the activation of back muscles required sufficient antagonistic moments to stabilize the upper body and or trunk. Hence, the reduction in vertical push-off could be a compensatory mechanism to alleviate back pain. As a result, the reduced vertical GRF in patients with CLBP may be due to the fear of exerting a push-off.

The asymmetry indices of loads applied on legs are shown in Table 5. There was no asymmetry of loads between legs in NCLBP. Therefore, it can be concluded that the loads transmitted through the legs cannot be a source of this pathology. Actually, there are two theories to explain the source of the pain and its relation with physical activity in subjects with NCLBP [26]. The first theory is known as fear avoidance model (FAM) and emphasizes the fact that NCLBP patients decrease their physical activities to reduce their pain. In contrast, the second theory, avoidance-endurance model (AEM), emphasizes that NCLBP patients ignore their pain and persist on moving around despite their pain [26]. As there was no difference between the loads applied on the legs and also symmetry of the loads between health subjects and patients with NCLBP, it can be concluded that these subjects try to not change their physical performance. It should be emphasized that asymmetry of loads on the right and left sides has been studied in some disorders including scoliosis and cerebral palsy, which also correlates with the severity of pathology.

Finally, there are limitations in this study. First, only the force applied on the legs was evaluated. Therefore, it is recommended that in the future studies the moment applied on the leg will also be evaluated. Secondly, no information regarding the site of pain in patients with LBP was collected in our study. Hence, it is difficult to say whether the activities of the lumbar muscles during the stance phase were associated with the site of pain. Lastly, a cross-sectional design was used in this study. The results from such design may not reflect the population studied because different observations may be obtained if the intervention was carried out in another time frame. Therefore, the clinicians are to interpret the findings of this study with caution. It is hoped that these limitations could be addressed in future studies

## 5. Conclusion

There were neither any differences between the forces applied on the leg between normal and NCLBP subjects nor any differences between asymmetry indices. Therefore, it can be concluded that NCLBP subjects follow avoidance-endurance model without any limitation during walking. It also can be concluded that the asymmetry of force cannot be a reason of low back pain.

## Figures and Tables

**Table 1:** The characteristics of NCLBP and control subjects who participated in the study.

Characteristics	Groups	
	NCLBP∗ (*n* = 20)	Control (*n* = 20)
Age (y)	41.56 ± 9.57	40.18 ± 8.55
Height (m)	158.81 ± 5.56	158.18 ± 5.74
Weight (kg)	61.68 ± 8.88	60.25 ± 6.38
Sex	Female	Female

Values are mean ± SD.

*Nonspecific low back pain.

**Table 2:** Inclusion and exclusion criteria for patients who participated in the study.

Inclusion criteria	Exclusion criteria
Female Age: 25–55 years Pain group: (i) History of NSCLBP ≥6-month duration (ii) Without peripheral pain referral (iii) Pain in the area from T12 to gluteal folds (iv) Moderate ongoing LBP (a) Average daily pain level—VAS > 2/10 (b) Experienced most days of the week (v) Mechanically induced localized LBP Control group: No history of spinal pain	(i) Specific diagnosis associated with LBP such as spondylolisthesis, disc prolapse, and inflammatory disorders (ii) Presence of other conditions affecting the spine including neurological or metastatic disease (iii) Any neurological deficit (iv) Any surgery involving the lumbar spine (v) Any diagnosed pelvic or abdominal pain disorder in the last 12 months (vi) Pregnancy or being less than six months after partum (vii) Any lower limb surgery in the last 2 years (viii) Current lower limb injury (ix) An inability to understand written or spoken Persian

**Table 3:** Spatiotemporal gait parameters.

Parameter	Velocity (cm/s)	Cadence (steps/min)	Stride length (cm)
CLBP	9.2 ± 1.3	97.7 ± 9	1.13 ± 0.093
Normal	9.53 ± 0.99	98.3 ± 7.1	1162.62 ± 0.77
*P* value	0.245	0.4	0.17

**Table 4:** The peaks of the forces applied on the leg (dominant side) in normal and NCLBP subjects.

Parameter	Fz1 (N/BW)	Fz2 (N/BW)	Fz3 (N/BW)	Fy (N/BW)	Fx1 (N/BW)	Fx2 (N/BW)
NCLBP	1.038 ± 0.099	0.86 ± 0.0398	1.106 ± 0.0356	0.089 ± 0.022	0.178 ± 0.03	0.055 ± 0.011
Normal	1.02 ± 0.0354	0.0857 ± 0.0389	1.13 ± 0.045	0.1004 ± 0.036	0.19 ± 0.0218	0.053 ± 0.016
*P* value	0.25	0.42	0.038	0.214	0.138	0.353

Values are mean ± SD. N: newton, BW: body weight, Fz: vertical force, Fy: mediolateral force, and Fx: anteroposterior force.

**Table 5:** The results of asymmetry analysis of spatiotemporal and force parameters of normal and NCLBP subjects.

Parameter	WS (%)	Cadence (%)	SL (%)	Fz1 (%)	Fz2 (%)	Fz3 (%)	Fy (%)	Fx1 (%)	Fx2 (%)
CLBP	3.8 ± 3.4	3.23 ± 2.62	1.54 ± 1.33	2.59 ± 1.89	17.12 ± 48.46	2.4 ± 2	16.73 ± 12.9	7.21 ± 6.3	27.74 ± 26.1
Normal	5.03 ± 4.6	5.88 ± 4.84	2.42 ± 1.5	3.88 ± 2.94	2.4 ± 2.3	2.48 ± 2.2	16.3 ± 8.2	5.59 ± 8.8	22.56 ± 28.9
*P* value	0.3	0.15	0.152	0.2	0.169	0.48	0.46	0.36	0.37

WS: walking speed, SL: stride length, Fz: vertical force, Fy: mediolateral force, and Fx: anteroposterior force.
